# An Ultra-Durable Windmill-Like Hybrid Nanogenerator for Steady and Efficient Harvesting of Low-Speed Wind Energy

**DOI:** 10.1007/s40820-020-00513-2

**Published:** 2020-08-27

**Authors:** Ying Zhang, Qixuan Zeng, Yan Wu, Jun Wu, Songlei Yuan, Dujuan Tan, Chenguo Hu, Xue Wang

**Affiliations:** 1 Department of Applied Physics, State Key Laboratory of Power Transmission Equipment and System Security and New Technology, Chongqing University, Chongqing, 400044 People’s Republic of China; 2 Chongqing Key Laboratory of Soft Condensed Matter Physics and Smart Materials, Chongqing University, Chongqing, 400044 People’s Republic of China

**Keywords:** Triboelectric nanogenerator, Windmill-like structure, Rotational contact-separation mode, Low-speed wind energy harvesting

## Abstract

Wind energy is one of the most promising and renewable energy sources; however, owing to the limitations of device structures, collecting low-speed wind energy by triboelectric nanogenerators (TENGs) is still a huge challenge. To solve this problem, an ultra-durable and highly efficient windmill-like hybrid nanogenerator (W-HNG) is developed. Herein, the W-HNG composes coupled TENG and electromagnetic generator (EMG) and adopts a rotational contact-separation mode. This unique design efficiently avoids the wear of friction materials and ensures a prolonged service life. Moreover, the generator group is separated from the wind-driven part, which successfully prevents rotation resistance induced by the friction between rotor and stator in the conventional structures, and realizes low-speed wind energy harvesting. Additionally, the output characteristics of TENG can be complementary to the different performance advantages of EMG to achieve a satisfactory power production. The device is successfully driven when the wind speed is 1.8 m s^−1^, and the output power of TENG and EMG can achieve 0.95 and 3.7 mW, respectively. After power management, the W-HNG has been successfully applied as a power source for electronic devices. This work provides a simple, reliable, and durable device for improved performance toward large-scale low-speed breeze energy harvesting.

**Electronic supplementary material:**

The online version of this article (10.1007/s40820-020-00513-2) contains supplementary material, which is available to authorized users.

## Highlights

- A novel windmill-like hybrid nanogenerator with contact-separation structure was proposed for harvesting breeze energy at low wind speed.
- A spring steel sheet was creatively used both as an electrode of triboelectric nanogenerator and a booster for contact-separation activity.
- A magnetic acting as a bifunctional element supplies magnetic flux variation in electromagnetic generator and overcomes electrostatic adsorption between tribolayers simultaneously.

**Electronic supplementary material:**

The online version of this article (10.1007/s40820-020-00513-2) contains supplementary material, which is available to authorized users.

## Graphical abstract

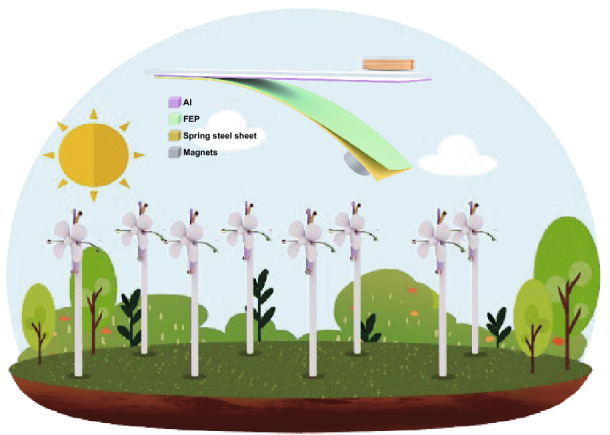

## Introduction

With the rapid development of the Internet of things, powering the ubiquitous and distributed sensors has become a key challenge [1–4], and converting ambient energy into available electricity is an attractive approach to solve this problem [5–8]. Among various types of energy resources in nature, such as solar, mechanical, tidal energies, and so on, wind energy is one of the most attractive candidates because it is a sustainable clean energy with the advantages of wide distribution and enormous reserves [9–12]. Therefore, extensive research has been carried out to develop efficient wind energy harvesting technologies. During the last decades, conventional wind turbine generators have been extensively used for converting wind energy into electricity based on the mechanism of electromagnetic induction [13, 14]. Nevertheless, the disadvantages of cumbersome structure, high manufacturing cost, and installation height of more than 40 meters [15, 16] severely restrict the widespread application of traditional wind turbine generators [17, 18]. Therefore, it is more and more urgent to develop alternative approaches for wind energy harvesting with a lower cost, easier maintaining, and higher efficiency in ordinary environments.

In recent years, the emerging triboelectric nanogenerator (TENG) based on coupling triboelectric effect and electrostatic induction [19–22] is considered as a promising mechanical energy scavenging and conversion technology. Benefiting from advantages of lightweight, materials variety, easy fabrication, and cost-effectiveness, TENG has been proven to be one of the most efficient ways for harvesting low-frequency mechanical energies such as human movements, wind energy, wave energy, and so on [23–26]. TENG will undoubtedly provide a new power supply manner for intelligent, wearable, and implantable electronic products. To date, some wind energy harvesting TENGs with rotational structures [27–30] and flutter-driven structures were developed [31–36], and quite high outputs could be achieved under relatively high wind speeds which were usually higher than 5 m s^−1^. However, the global average wind speed near the surface (the observation altitude is 10 meters) is reported to be 3.28 m s^−1^ [6, 37]. Therefore, this factor severely limits the widespread applications of the previously reported TENG-based wind energy harvesters in practice. Moreover, friction layers of these conventional wind energy acquisition devices usually suffered destructive friction wear caused by the rotation-sliding working mode or direct contact with wind, which led to a reduction in device service life. So, it is of great significance to design a new invention to solve the above problems.

In this work, we present a novel windmill-like triboelectric–electromagnetic hybrid nanogenerator (W-HNG) to harvest low-speed wind energy. The nanogenerators are mounted on a cross-shaped frame and connected with a small fan. When the wind blows, the fan will take the generator group to demonstrate a rotational movement, and benefiting from the unique design, the rotational motion will be transformed into a contact-separation activity and the generators will start to work. This creative design can effectively harvest wind energy and avoids huge rotation resistance and friction wear extensively existed in the conventional TENGs with rotary configurations. What’s more, a spring steel plate is creatively utilized in this W-HNG which plays a dual role: One is playing as an electrode of TENG, which simplifies device fabrication process. The other is acting like an accelerator, because it can store elastic potential energy and converts it into kinetic energy, which can boost the contact-separation velocity and improve contact strength between the two friction materials. Additionally, the magnet in this W-HNG is also a bifunctional element. One is to supply magnetic flux variation in EMG to generate electricity, and the other is acting as an additional weight to overcome the electrostatic adsorption between tribolayers and facilitates the contact-separation activities. After systematical structure design and optimization, this W-HNG can effectively collect airflow energy in light breeze ranging from 1.8 to 4.8 m s^−1^, and the practical applications as a sustainable power supply are also demonstrated. This work may give new insights to solve the recent problems of TENG-based wind energy harvesters and provides a novel idea for breeze energy harvesting.

## Experimental Section

### Fabrication of TENG

First, two acrylic boards with a thickness of 4 mm and width of 2.5 cm were fabricated by laser cutting technique and selected as the substrate material, and the length was altered (16, 20, 24, 28, and 32 cm) to match the varied electrode sizes. In each acrylic board, a groove (12.5 × 4 mm^2^) was created in the middle, so that two acrylic plates could be bonded together to form a cross-framework. Then, four pieces of Al foils (15 μm in thickness) were selected as one electrode for TENG component and attached onto the four arms of the acrylic framework in a clockwise order. After that, a spring steel sheet with a width of 2.5 cm was chosen as the other electrode material as well as a substrate for FEP film (with glue on the backside and 30 μm in thickness), and the lengths of spring steel sheet and FEP film were adjusted (6, 8, 10, 12, and 14 cm) to realize the device structural optimization. After assembling, the FEP/spring steel sheet was mounted face to face with the Al electrode when keeping the FEP layer in the inner side. After that, a fan (8 cm in diameter) was utilized for wind energy acquisition and connected with the hybrid nanogenerator by a straight acrylic rod (8 mm in diameter and 10 cm in length). For comparison, a disk-type TENG (10 cm in diameter) adopting rotational sliding mode was fabricated, which employed Al foil as the electrode material and FEP film as the dielectric layer.

### Fabrication of EMG

Each EMG unit consists of two parts: a custom-made copper coil (0.015 mm in wire diameter, 2500 in turns) and a group of cylindrical magnets. First, the magnets were fixed precisely on the free tip of spring steel sheet (each magnet was 2 mm in thickness, 12 mm in diameter, and 1 g in weight), and the number of magnets could be readily adjusted for realizing an optimal contact-separation movement and ideal output. Then, the copper coil was adhered on the tip of acrylic arms and located at the backside of Al electrode. After that, a small fan was connected to the middle of the acrylic framework acting as the wind scavenging equipment.

### Electrical Measurement

The output signals of TENG and EMG were acquired via a programmable electrometer (Keithley 6514 System Electrometer). The software platform was built based on LabVIEW, which was capable of realizing real-time data acquisition and analysis. Comsol Multiphysics software based on finite element simulation was used to calculate the potential distribution of TENG unit under open-circuit condition. For simulation, the relative dielectric constants of steel sheet and Al were set as infinity, while that of FEP was set as 1. Additionally, the lengths of the three materials are assumed as 200 mm and the heights are 0.5 mm. A stepper motor was applied to provide a periodic rotation and precisely adjusted the frequencies in the experiments. The output performances of the W-HNG associated with real wind were measured under simulated air flow produced by an electrical fan, and a commercial anemometer (UNI-T UT363BT) was applied to measure the wind speeds.

## Results and Discussion

### Structural Design and Working Mechanism

**Fig. 1:**
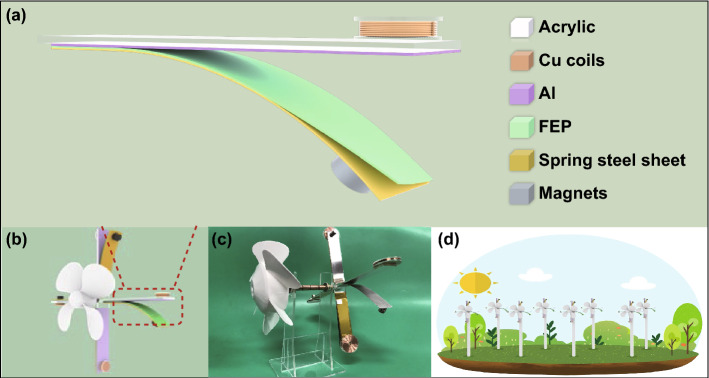
Schematic illustration of the windmill-like hybrid nanogenerator (W-HNG). **a** Structural scheme of an individual unit of the W-HNG. **b** Schematic illustration of the whole W-HNG. **c** Photograph of the as-fabricated W-HNG. **d** Proposed network composed of numbers of W-GNGs for harvesting large-scale low-speed wind energy

As shown in Fig. 1, a typical W-HNG is assembled by four individual hybrid nanogenerators, and every single unit consists of two parts: a TENG and an EMG. The structural design of a single hybrid nanogenerator is schematically outlined in Fig. 1a. As can be seen, a contact-separation mode TENG cell is attached on the bottom side of an acrylic substrate. Due to the specific elasticity and conductivity, a spring steel sheet is creatively used here both as an electrode of TENG and as a support to allow the tribolayer (a fluorinated ethylene propylene (FEP) film) sticking on its top surface. Besides, there is an Al foil adhered to the acrylic plate serves as the other electrode and friction layer. As we know, after several contacts, great electrostatic attraction force will generate between Al and FEP film, and the two electrodes will stick together tightly and contact-separation motion will suspend. To avoid that, a magnet is introduced to adhere on the free tip of spring steel sheet, which plays a dual role: One is an additional weight to facilitate the contact-separation activity between Al and FEP, while the other is the magnetic source of EMG, and the rest part of EMG is a copper coil fixed on the top tip of the acrylic plate. After assembling, four independent hybrid nanogenerators with the same structure are arranged along with a clockwise order and mounted on the frame with a fan, and the schematic diagram and digital picture of a holistic W-HNG device are, respectively, depicted in Fig. 1b, c. Relying on the advantages of easy fabrication, low cost, lightweight, and high efficiency, this W-HNG can be integrated into networks and widely installed in the open space for large-scale wind energy harvesting, as illustrated in Fig. 1d.

**Fig. 2:**
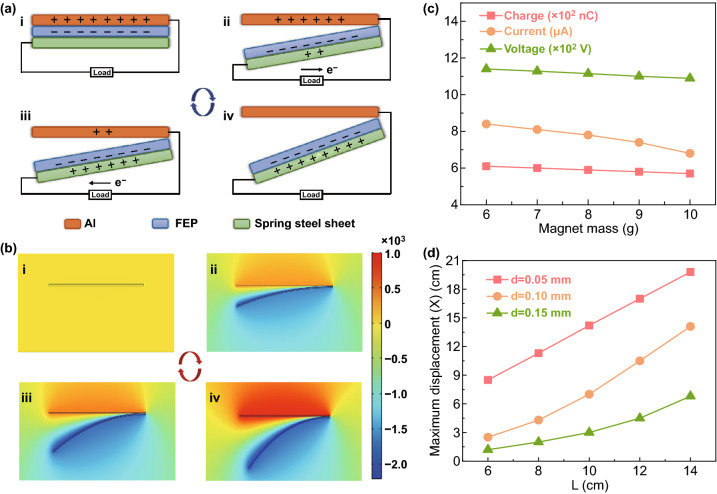
**a** Working mechanism of the W-HNG. **b** Potential distribution across the W-HNG electrodes under open-circuit conditions at different angles states, as evaluated by COMSOL. **c** *Q*_sc_, *I*_sc_, and *V*_oc_ signals under diverse magnet mass. **d** Varied maximum displacement between FEP and Al on changing both the thickness and length of the spring steel sheet

When the small fan is subject to wind, it will take the nanogenerator group connected behind rotating together, and the nanogenerators will take turns to demonstrate periodical contact-separation motion. The electric generation mechanism of the W-HNG can be divided into two parts: TENG and EMG. For the TENG components, based on the coupling effect of triboelectrification and electrostatic induction [38, 39], the working mechanism can be illustrated in four consecutive steps in a full cycle as shown in Fig. 2a. Initially, after the first contact (state i), electrons will transfer from the Al foil to the FEP film since the latter is much more electronegative than the former [40, 41]. Then, the generator is triggered by wind to rotate forward and two tribolayers will separate from each other (state ii). Because of the electrostatic induction, positive charges will transfer from the Al electrode to the spring steel plate through the external circuit to balance the electric field, thus generating a pulse current. When the FEP film is separated from the Al electrode to the maximum displacement (state iii), the positive charges on Al electrode will be completely neutralized by induced electrons from steel sheet. After that, as the device continues to rotate, the steel sheet will gradually move back to the Al foil until they are fully overlapped again (state iv), and a current in the opposite direction will be produced. Consequently, alternating current (AC) output would be achieved with the device rotation. To demonstrate the working mechanism more clearly, the corresponding electric potential distribution in vacuum under an open-circuit condition was simulated via the finite element method using COMSOL multiphysics software, and the results are illustrated in Fig. 2b. Meantime, based on the electromagnetic induction [42, 43], the EMG part can produce an alternating current due to the periodic change of magnetic flux caused by cyclical displacement variation between magnet and copper coil.

### Structural Optimization

To quantitatively evaluate the output performance of W-HNG, the device was mounted on a stepper motor which could generate controllable rotation with a tunable speed. First, in order to get optimal output performance of the TENG part, the influence of different structure parameters was experimentally tested under a constant rotation velocity of 15 rpm while the width of steel sheet was fixed at 25 mm. We initially fabricated a device without magnet fixed on the steel sheet tip, and the electrical output performance is illustrated in Fig. S1. Obviously, with this design, output signals of the transfer charge quantity (*Q*_sc_), short-circuit current (*I*_sc_), and open-circuit voltage (*V*_oc_) are too weak to provide available electricity. The key reason causing this phenomenon was that the strong electrostatic attraction between Al electrode and PTFE film hampered effective contact-separation activity. To solve this problem, a magnet was introduced to be fixed at the steel plate tip, which not only served as an external load to promote the contact-separation motion between two friction materials, but also played as the magnetic source of EMG. By testing, it was observed that the spring steel sheet and Al foil could be well separated when the additional magnet mass reached 6 g. However, the output performance of TENG decreased gradually with the increase in magnet weight, especially the current, as shown in Figs. 2c and S2, which could be ascribed to the fact that a heavier magnet promoted separation but prevented contact of the two electrodes. Therefore, magnets of 6 g were chosen for the next tests.

**Fig. 3:**
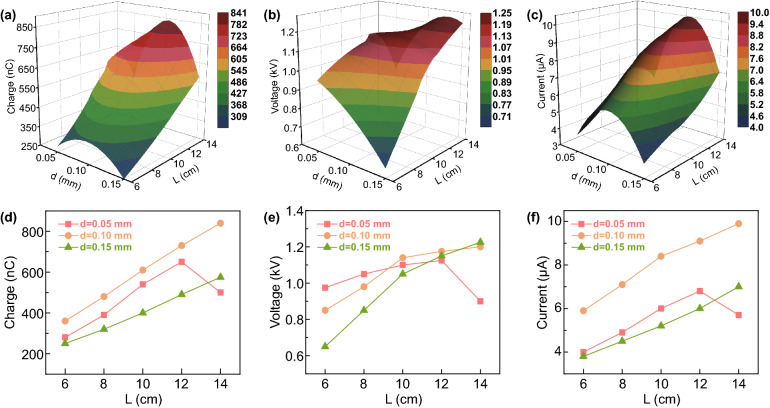
W-HNG configuration optimization. 3D surface graphs of **a** *Q*_sc_, **b** *V*_oc_, and **c** *I*_sc_ on changing both the thickness and length of the spring steel sheet and their corresponding 2D graphs (**d**–**f**)

It is obvious that steel band stiffness shows a significant impact on the contact/separation status of two electrodes, which is closely related to the output performance of TENG [44–46]. Therefore, steel plate with different thicknesses and lengths was studied for the optimum structural design. As shown in Fig. 2d (the specific annotation of each parameter is shown in Fig. S3), at a stationary state, a larger maximum displacement (*x*) between Al foil and FEP film can be achieved at a longer and thinner steel plate. In other words, steel plate with smaller stiffness is beneficial to the separation of two electrodes, which may be favorable for a high output voltage according to the formula as bellows [47]:

1 $$V = - \frac{Q}{{S\varepsilon_{0} }}\left( {d_{0} + x\left( t \right)} \right) + \frac{\sigma x\left( t \right)}{{\varepsilon_{0} }}$$

where *Q* defines the amount of transferred charges between the two electrodes, *S* is the effective contact area between the tribolayers, *d*_0_ is the dielectric thickness, *ε*_0_ denotes the vacuum dielectric constant, *x*(*t*) is the separation distance between two friction materials, and б is the triboelectric charge density. However, at a rotational status, the steel sheet stiffness is not the smaller the better, because it will directly affect the contact strength, effective friction area, and contact/separation velocity of two electrodes, thus influencing the electrical production of TENG [48–50]. Therefore, it is of great significance to figure out the optimal steel plate parameter. Electric output performance of TENG under various steel belt lengths and thicknesses has been measured at a rotation speed of 100 rpm, as illustrated in Fig. 3, where all 3D graphs (Fig. 3a–c) are smoothed by bilinear interpolation algorithm to be easily understood, and their corresponding 2D graphs are exhibited in Fig. 3d–f. As depicted in Fig. 3a, d, according to the *Q*_sc_ signals of steel sheets with 0.05, 0.10, and 0.15 mm thickness, it can be concluded that the transferred charge quantity is proportional to the effective contact area between two friction materials, which is corresponding to the literatures [51, 52]. However, there is an abnormal point of 0.05 mm steel belt at a length of 14 cm. The reason causing this phenomenon is that the stiffness coefficient of the steel sheet is too small to restore its original state during rotating, thus resulting in a reduced contact area between friction materials. Moreover, an optimal *Q*_sc_ was achieved at 0.10 mm thickness, revealing that the steel belt should be neither too flexible nor too stiff, as too much flexibility will reduce the force that it exerts on the Al electrode according to Hooke’s law *F *= *KX* (where *K* denotes the spring stiffness coefficient and *X* is the degree of deformation), and too much stiffness results in an inadequate contact between the tribolayers. The results of output voltage (measured by a voltage division method, as illustrated in Fig. 3b, e and short-circuit current (Fig. 3c, f) reveals that optimal performance can be realized at steel plate with 0.10 mm thickness, which is consistent with the results of *Q*_sc_. It is worth noting that both of *V*_oc_ and *I*_sc_ were increased with the growth of effective working area, but a slower pace appeared when the length exceeded 10 cm. Therefore, a steel sheet with 0.10 mm in thickness, 10 cm in length, and 25 mm in width was defined as the optimal structure parameter for the following measurements and demonstrations.

### Performance for Wind Energy Harvesting

**Fig. 4:**
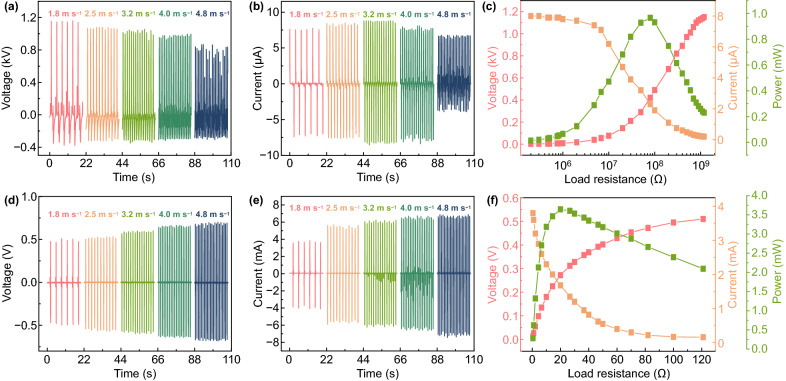
Electrical output performance of the W-HNG. The *V*_oc_ and *I*_sc_ of an individual TENG (**a**, **b**) and EMG (**d**, **e**) at wind speed ranging from 1.8 m s^−1^ to 4.8 m s^−1^. *V*_oc_, *I*_sc_, and power dependence of the external load resistance of the TENG **c** and EMG **f** at a wind speed of 1.8 m s^−1^

To further study the output of W-HNG, electrical performances of individual TENG and EMG were characterized under different wind speeds ranging from 1.8 to 4.8 m s^−1^, and results are depicted in Fig. 4. As shown in Fig. 4a, b, the peak value of the voltage for TENG is inversely proportional to wind speed, varying linearly from 1150 to 860 V, while the *I*_sc_ of TENG demonstrates a parabolic trend with a maximum value of 8.5 μA when the wind speed is 3.2 m s^−1^. According to the previous study [19], peak value of the output voltage is independent of the operation frequency, but it dependents on the displacement between two friction materials as expressed in Eq. 1. Because of this, a linear decay of the voltage signal is observed, and the reason is the relative displacement (*x*) between two friction layers gradually decreases with the raise wind speed. However, the *I*_sc_ is closely related to operation frequency which is proportional to the wind speed. Therefore, the measured *V*_oc_ and *I*_sc_ of TENG are consistent with the theoretical results. Moreover, in a TENG system, the device durability is extremely important for its practical application and indeed dependent on the working mode. To demonstrate the superiority of this novel configuration, the output signal of *Q*_sc_ was examined under wind speed of 1.8 m s^−1^ for a long-term monitoring. The results manifest that the output performance of the TENG does not decay significantly (decreases by ~ 2%) after 5 days of operation as outlined in Fig. S4, implying that it is an efficient way that transforming rotary movement into a contact-separation behavior to ensure a prolonged device service life as well as stable electrical output performance. To further display the advantages of the W-HNG, we designed a comparative experiment to compare the electrical output performance. As shown in Fig. S5, the transferred charge quantity of a disk-type TENG based on rotational sliding mode is decreased by 50% from 90 to 45 nC after 40 min of continuous operation. Moreover, since the tribolayer surface plays an important role in the triboelectric performance, microstructures of FEP film before and after durability test were characterized by SEM and results are shown in Fig. S6. Obviously, the friction wear of W-HNG is much smaller than the disk-type TENG, which further implies the designed superiority of W-HNG.

For EMG, both the *V*_oc_ and *I*_sc_ are proportional to the wind speed and increase from ~ 0.5 to ~ 0.7 V and from ~ 3.7 to ~ 6.7 mA, respectively, as shown in Fig. 4d, e. According to Faraday’s law, the open-circuit voltage and the short-circuit current of EMGs can be expressed as [53]:

2 $$V_{\text{OC}}^{\text{EMG}} = - N\frac{{{\text{d}}\emptyset }}{{{\text{d}}t}}$$

3 $$I_{\text{SC}}^{\text{EMG}} = \frac{{V_{\text{OC}}^{\text{EMG}} }}{R}$$

where $\emptyset$ is the magnetic flux and *N* is the number of turns in the copper coils. *R* is the internal resistance of the coil. Based on Eq. 2, the output voltage of EMG is strongly related to the variation rate of the magnetic flux. Therefore, the *V*_oc_ and *I*_sc_ of EMG should be proportional to the operation wind speed, consistent with the experimental results.

Further, to evaluate the energy output capability of W-HNG at a low wind speed, the output power of TENG and EMG when connected to different external load resistances was measured at a wind velocity of 1.8 m/s, as shown in Fig. 4c, f. It is found that whether for TENG or EMG, the output voltage increases as the resistance grows, and due to ohm loss, the short-circuit current follows a reverse trend. The maximum output power can be calculated as 0.95 mW for TENG and 3.7 mW for EMG at the corresponding matched load. Since the energy generation characteristic of TENG is totally distinct from EMG, the TENG can play as a voltage source while EMG can act like a current source.

### Practical Applications

**Fig. 5:**
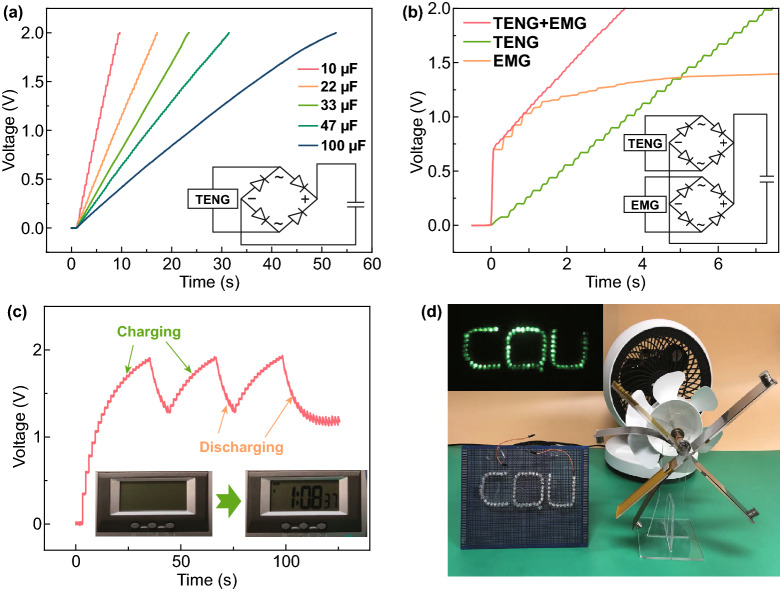
Applications of the W-HNG. **a** Voltage curves of several commercial capacitors charged by the TENG harvester at a wind speed of 4.8 m s^−1^, and inset is the charging circuit diagram. **b** Charging curves of a 10 μF capacitor using TENG only, EMGs, and the hybrid nanogenerator, and the inset is the rectifier circuit diagram. **c** Charging voltage profile of a 100 μF capacitor for several consequent working cycles of a digital watch at a wind speed of 1.8 m s^−1^. **d** Photograph of W-HNG acting as the power source for lighting LEDs

For the purpose of practical applications, the capability of this novel W-HNG for harvesting wind energy and powering electric devices are demonstrated in Fig. 5. First, as shown in Fig. 5a, the charging capability of an individual TENG was investigated at a wind speed of 4.8 m s^−1^, and a rectification circuit was used to convert AC signals into DC outputs as illustrated in the inset. As can be seen, the TENG took different amounts of time to charge capacitors varying from 10 to 100 μF to 2 V, where a 10 μF capacitor could be charged to 2 V after 7.5 s while a 100 μF capacitor needed 52 s. After that, the charging capability of the W-HNG was explored and compared with that of an individual TENG and EMGs (four in series), as illustrated in Fig. 5b, where a capacitor of 10 μF was used for measurement. Apparently, the charging rate of EMGs is faster than that of TENG at the beginning due to the high output current characteristic, but the voltage quickly gets saturated (only reaches 1.25 V). In contrast, the W-HNG provides not only high charging voltage but also fast charging speed that the capacitor can be charged to 2 V only in 3.5 s. Then, a digital watch driven by W-HNG was demonstrated by utilizing a 100 µF commercial capacitor to store the electric energy at a wind speed of 1.8 m s^−1^. As illustrated in Fig. 5c and Video S1, while the wind blows the W-HNG, the capacitor can be charged and the digital watch gets to work when the voltage rises to 1.9 V, and the device can be continuously powered as long as the W-HNG works. Moreover, when the wind speed is 1.8 m s^−1^, 70 LEDs can be continuously lighted up by W-HNG, as shown in Fig. 5d and Video S2. These results imply that the W-HNG can be used not only to harvest wind energy, but also to realize a self-powered system.

## Conclusion

In summary, we have developed a novel hybrid nanogenerator based on a windmill-like structure. Benefiting from the rotational contact-separation working mode, the W-HNG demonstrates a superior robustness and can be used to harvest low-speed wind energy with an excellent output performance. Relationship between the electrical properties of W-HNG and various structural parameters is systematically studied under different wind speeds. By optimizing the device structure, the maximum output voltage of the designed TENG unit can reach 1150 V, and the maximum current of the EMG unit can reach 6.7 mA. The capability of this device for practical applications such as driving LEDs, powering commercial capacitors, and driving electronic watch by harvesting wind energy was also demonstrated. Because of the lightweight, easy fabrication and portable merits, the W-HNG provides an effective way to capture breeze energy for driving low-power portable electronic devices.

## Electronic Supplementary Material

Below is the link to the electronic supplementary material.

Supplementary material 1 (PDF 5178 kb)

Supplementary material 2 (WMV 9967 kb)

Supplementary material 3 (WMV 3552 kb)
