# Supplementary material for: An Ultra-Durable Windmill-Like Hybrid Nanogenerator for Steady and Efficient Harvesting of Low-Speed Wind Energy
Source: Nanomicro Lett. 2020 Aug 27;12:175. doi: 10.1007/s40820-020-00513-2 (PMC7770936; doi:10.1007/s40820-020-00513-2)
Supplement: Supplementary file 1 — Supplementary material 1 (PDF 5178 kb) [file 40820_2020_513_MOESM1_ESM.pdf]

Supporting Information for

An Ultra-Durable Windmill-Like Hybrid Nanogenerator for Steady and Efficient Harvesting of Low-Speed Wind Energy

Ying Zhang[1], Qixuan Zeng[1], Yan Wu[1] , Jun Wu[1], Songlei Yuan[1], Dujuan Tan[1], Chenguo Hu[1, 2], Xue Wang[1, 2, *]

[1]Department of Applied Physics, State Key Laboratory of Power Transmission Equipment & System Security and New Technology, Chongqing University, Chongqing 400044, People's Republic of China

[2]Chongqing Key Laboratory of Soft Condensed Matter Physics and Smart Materials, Chongqing University, Chongqing 400044, People's Republic of China

*Corresponding author. E-mail: xuewang@cqu.edu.cn (Xue Wang)

Supplementary Figures

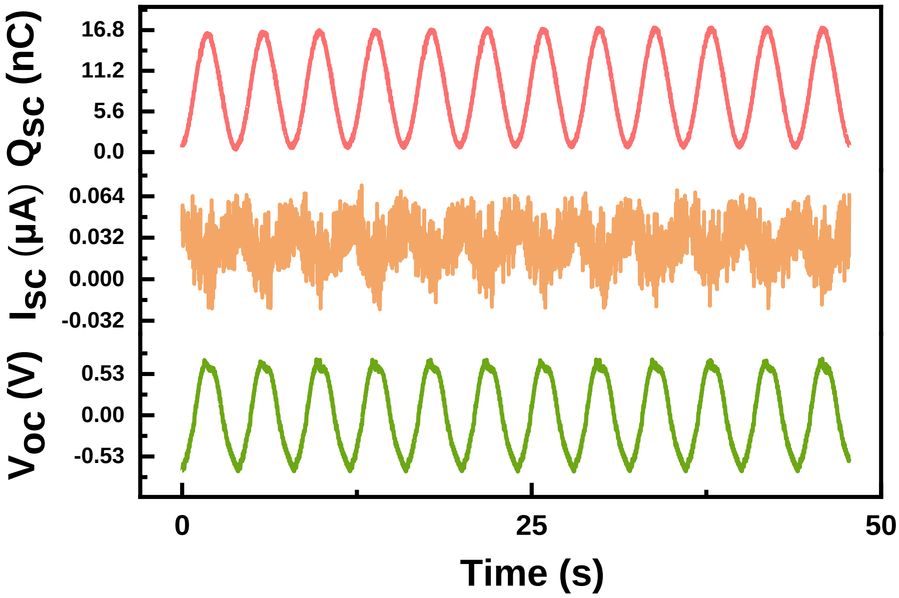

Fig. S1 Electrical output performance of W-HNG without magnet at rotation speed of 15 rpm

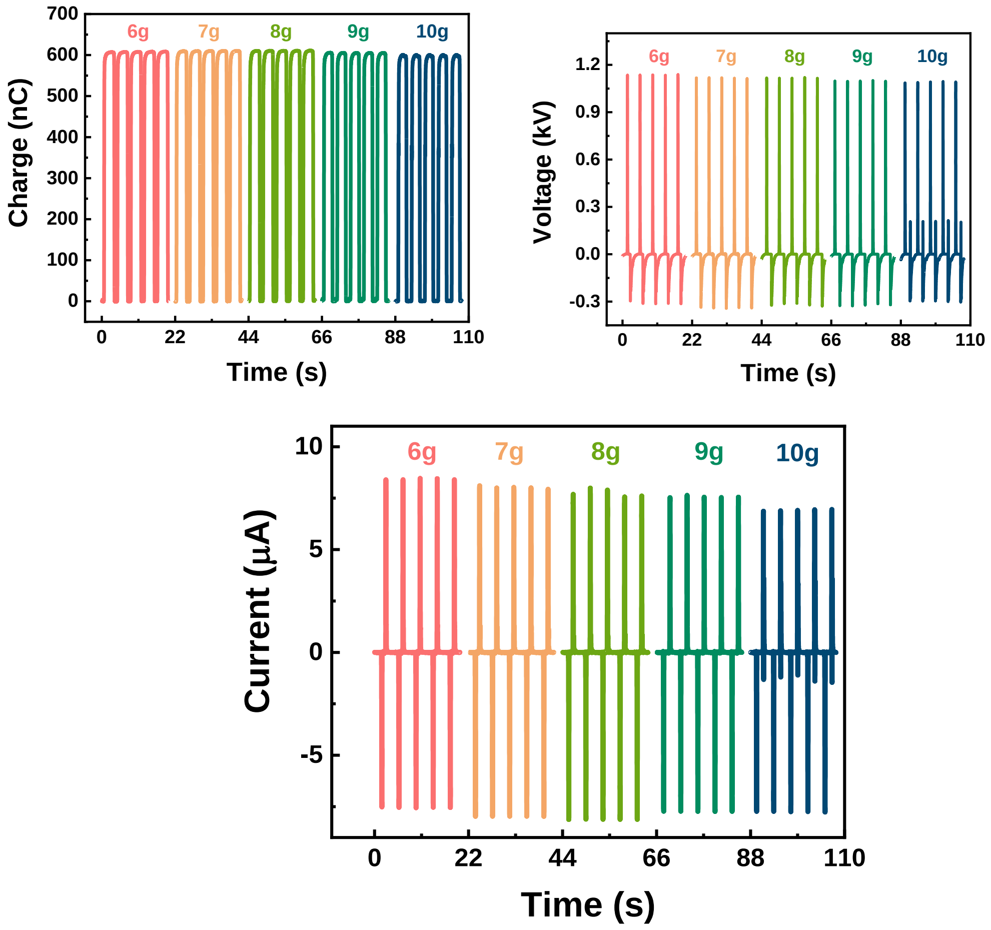

Fig. S2 Electric output signals of the TENG component with different magnet mass at the rotation speed of 15rpm

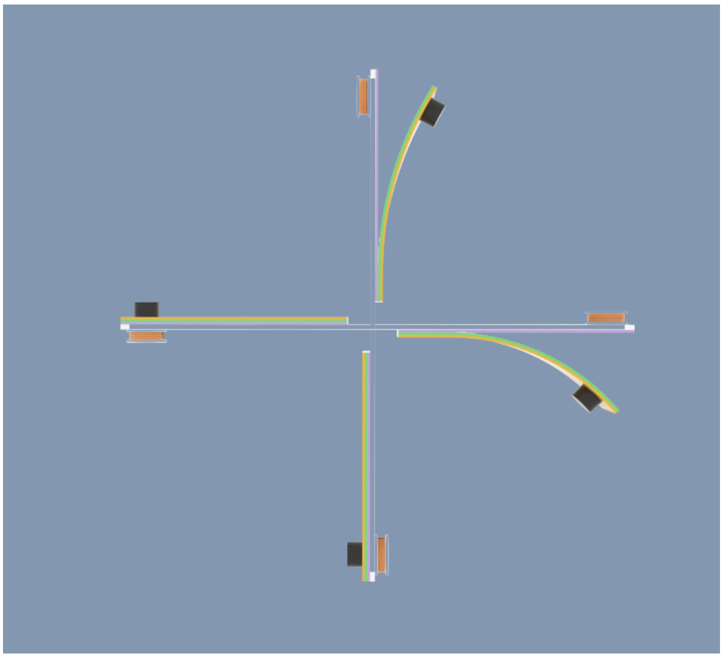

Fig. S3 Annotation of specific parameter of the device

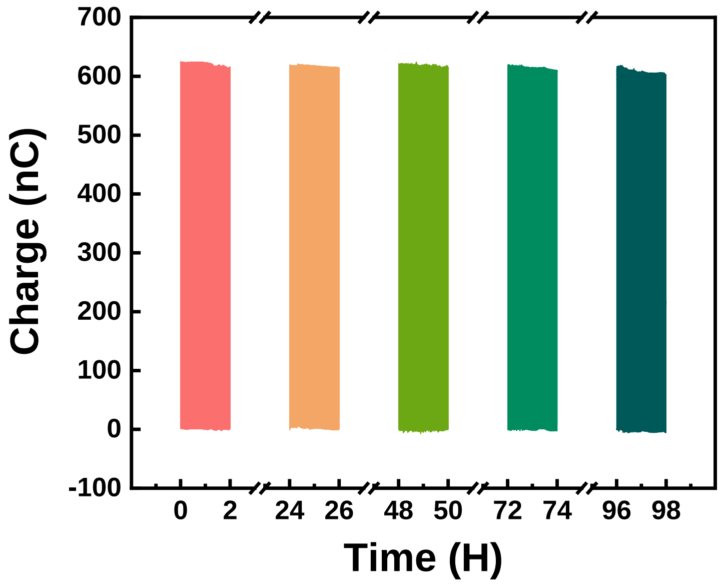

Fig. S4 Durability and electrical stability test of W-HNG for continuous operation of 5 days at a rotation speed of 15rpm

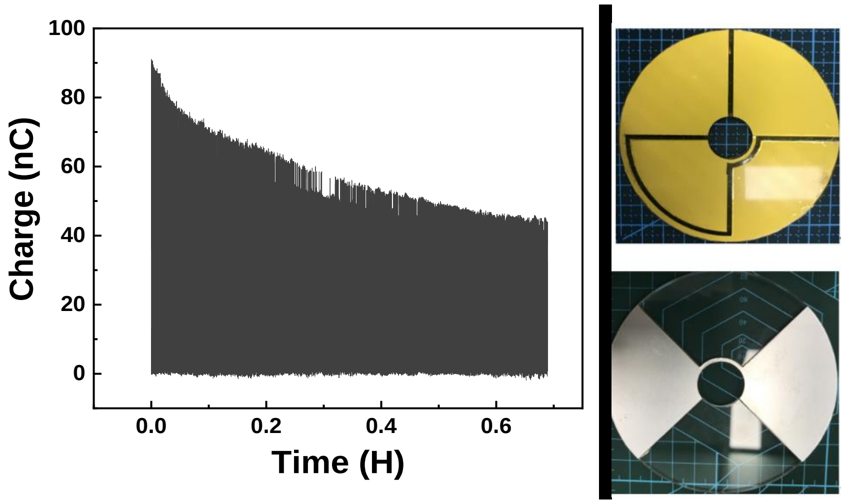

Fig. S5 Digital pictures and durability test of a disk-type triboelectric nanogenerator at a rotation speed of 15rpm

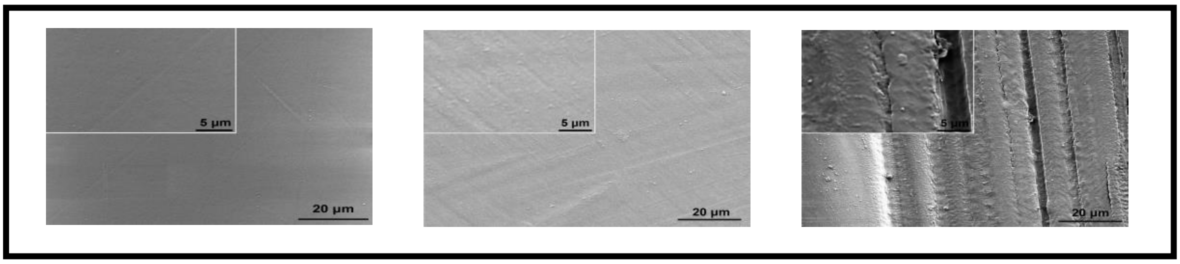

Fig. S6 SEM pictures of FEP films: (**a**) a pristine one, (**b**) W-HNG, and (**c**) disk-type TENG after five days durability test
